# Mentoring Nurses in Political Skill to Navigate Organizational Politics

**DOI:** 10.1155/2016/3975634

**Published:** 2016-09-29

**Authors:** Wanda Montalvo, Mary W. Byrne

**Affiliations:** ^1^Weitzman Institute, Middletown, CT, USA; ^2^Columbia University School of Nursing and College of Physicians and Surgeons, New York, NY, USA

## Abstract

*Objective*. The aim of this study was to describe and analyze the correlations between mentoring functions and political skill development among nurses who have earned or are candidates for a Ph.D. or doctorate of nursing practice (DNP) degree.* Background*. The healthcare system is in flux; future generations of Ph.D. and DNP nurse leaders will be required to demonstrate political acumen. Political skill to navigate organizational politics has had limited research within nursing.* Methods*. A cross-sectional research design using a web-based survey of 222 nurses who have earned or are candidates for a Ph.D. or DNP. This study utilized two validated tools to measure mentoring functions and political skill.* Results*. The response rate was 52% (*n* = 115) of which 86 were Ph.D. and 29 were DNPs. An informal mentoring relationship was described by 62% of the respondents and formal mentoring by 35% of the protégés; only 25% (*n* = 74) established a mentoring contract. Mentoring score showed significance for total political skill and moderate effect on the networking ability. The mentoring functions of advocacy, career development facilitation, learning facilitation, and friendship were found to correlate significantly with total political skill scores.* Conclusions*. This study established a benefit for nurses who have earned or are candidates for a Ph.D. or DNP from mentoring to support political skill development.

## 1. Introduction

The evolving healthcare system is poised to benefit from having highly educated nurses leading improvements in healthcare quality, evidence-based practice (EBP), and research. Nurses must possess sufficient political skill to successfully navigate organizational structures and relationships and produce results in these complex systems.

Political skill is distinct from policy making ability in that the skill is related to a purposeful strategy to enhance job performance within the context of the organizational environment [1–5] and not government or agency policy [4]. Political skill is defined by Ferris as “the ability to effectively understand others at work, and to use such knowledge to influence others to act in ways that enhance one's personal and/or organizational objectives” [2, p. 7]. Political skill is limited as a topic in the nursing literature but it is studied extensively in organizational psychology [4]. Political skill has been identified as an essential skill for nurse leaders to effectively influence others, navigate organizational politics, and enhance career advancement.

Nurses with either an earned degree or candidacy for doctorate in philosophy (Ph.D.) or doctorate of nursing practice (DNP) represent highly educated nurses who are trained to approach issues including political savvy through scholarship. These nurses will be expected to fill leadership roles in both academics and practice, requiring high level political skills to navigate complex environments and organizations. The Ph.D. prepared nurse helps educate future generations of nurses and conducts research to improve nursing practice and health [5]. The Ph.D. nurse also assumes leadership and administrative roles in the healthcare system as well as in academic settings. DNP prepared nurses are clinical scholars with the capacity to translate research, shape systems of care, and influence organizational-level research to improve performance using informatics and quality improvement models [5]. Both Ph.D. and DNP prepared nurses make significant contributions and are essential for leading systems and organizations.

This paper reports results of a study that describes and analyzes the correlations between mentoring functions and political skill development among nurses who have earned or are candidates for a Ph.D. or DNP. Such nurses may excel without a mentor, but having a mentor can assist the protégé in gaining access, momentum, exposure, and visibility within the organizational setting to accomplish their goals [6, 7]. It is hypothesized that mentoring functions are significantly related to political skill development. As emerging nurse leaders, both types of doctoral-prepared nurses benefit from developing a systems-perspective of healthcare in leading interdisciplinary teams across diverse delivery settings [8, 9].

## 2. Mentorship

The acquisition of leadership and political skill is refined by experiences preferably in the context of a mentor/protégé relationship. A protégé is defined as a less proficient or uninitiated individual selected as a beneficiary to receive counsel and affirmation by the mentor [7]. The protégé can garner benefits from mentoring because experience and contextual knowledge are guided through the relationship [3]. Mentors guide protégés in understanding the formal and informal power structures of an organization, an imperative for supporting emerging leaders. Absence of this ability puts the next generation of nurse leaders at risk to fail [10]. Mentoring is one mechanism to help expand and support development of nurse leaders and is endorsed by the Institute of Medicine (IOM) Future of Nursing Report [5]. Mentoring is an interpersonal relationship that requires mutual commitment from both the mentor and protégé to help facilitate positive career development [11].

## 3. Mentoring Contracts

The clarification of roles and expectations in the mentor-protégé relationship is important. A mentoring contract acts as a tool to help define the learning objectives of the protégé's professional and career goals [12]. Protégés expect mentors to help in the development of critical thinking skills, socialization to institutional culture, understanding of promotional requirements, and advocacy on behalf of the protégé [12]. A mentoring contract holds both the mentor and protégé accountable to agreed goals based on a timeframe that can be reevaluated or adjusted or conclusion of the mentoring relationship [13].

## 4. A Predicted Gap in Nursing Leadership

The IOM Future of Nursing Report made several strategic recommendations to help advance healthcare for all Americans including expanding the leadership roles of nurses [5]. Future generations of nurse leaders will be required to (a) understand power structures [14], (b) work with and lead interdisciplinary teams, (c) help educate a growing nursing workforce [8, 15, 16], and (d) understand the contextual factors impacting research and practice transformation [1]. A 2013 American Organization of Nurse Executives (AONE) study projected a 41%–67% turnover of chief nursing officers (CNOs) within the next three to five years [17]. While the 2013 AONE Position Statement acknowledges the need for executive nurse leaders prepared at the doctoral level, the minimal requirement is a master education in nursing [18]. Due to the paucity of data regarding the nursing leadership, research indicates a future deficit of 20% in the numbers of nurses below the projected requirements [19]. To offset the projected deficit of nurse leaders, we need more highly educated nurses who also possess sufficient political skill to manage complex work environments and reduce job stress [20].

## 5. Contemporary Mentoring Functions

In this study, contemporary mentoring functions [21] were utilized and defined as follows: (a) personal and emotional guidance; (b) coaching; (c) advocacy, (d) career development; (e) role modeling; (f) organizational systems advice; (g) learning facilitation; and (h) friendship. Mentoring as an interpersonal relationship requires mutual commitment of mentor and protégé to facilitate positive career development [11, 22–25]. In addition, similarities between mentor and protégé, such as personal values, communication, or work style, were considered because, as an interpersonal relationship, these can potentially influence the protégé perception of mentoring benefit [22, 26–28].

## 6. Methods

A cross-sectional research design was selected using a web-based survey. The mentoring frameworks utilized were informed by Yoder's [29] concept analysis of mentoring and Kram's Phases of Mentoring [30] which capture key variables that influence the mentoring relationship, including gender, age, career stage, ethnicity, and shared traits between mentor and protégé. The selected participants were scholars of the Jonas Center for Nursing and Veterans Healthcare (http://www.jonascenter.org/), a foundation which provides institutional awards to schools of nursing to support doctoral education for Ph.D. and DNP nurses. Participants for this study were Jonas Nurse Scholars (JNS) in Cohorts 1, 2, and 3 (*n* = 249) between the years of 2008 and 2014 who either have earned or are candidates for a doctoral degree. Exclusion criteria were newly selected JNS as of September 2014, non-JNS, or withdrawal from doctoral program. The Columbia University Institutional Review Board approved this study. Columbia University's Qualtrics Survey Research Suite, an online secure survey software, was utilized to administer the following surveys: Mentoring Functions Survey (MFS) [21, 31] and Political Skill Inventory (PSI) [32].

### 6.1. Sample Size and Recruitment

The sample size needed to measure the strength of correlation for a moderate effect of .30 with an alpha of 0.05 and power of 0.80 is 84 [33]. The participants selected were only the protégés, not the mentors, using a direct email with an imbedded 90-second introductory video explaining the study purpose and rationale, along with instructions for completing the survey using an embedded link. To increase response rate, one gift card was awarded at random following weeks 1, 2, and 3. Direct email reminders were sent to nonresponders on days 5, 15, and 25.

### 6.2. Description of Instruments

Structure, content, scoring, and internal consistency can be described for the two validated instruments: Mentoring Functions Survey (MFS) [21, 31] and Political Skill Inventory (PSI) [32]. The MFS is a 36-item instrument using a 6-point Likert scale (with responses ranging from “never” to “very frequently”) and has a reported Cronbach alpha of .93 [21]. In this study, the Cronbach alpha was .96. A sample question is “*mentor provided strategic advice on how to handle people or situations.*” The PSI is an 18-item instrument with extensive research use in organizational psychology but with minimal use in nursing [32, 34–38]. The PSI uses a 7-point Likert scale (with responses ranging from “strongly disagree” to “strongly agree”) and has a reported Cronbach alpha of .90 [32]. In this study, the Cronbach alpha was .89. A sample question is “*I am good at building relationships with influential people.*” The PSI consists of 4 subscales: social astuteness, interpersonal influence, networking ability, and apparent sincerity [32, 34–38]. The PSI mean scores indicate the following levels of political skill: low (1-2), average (3–5), and high (6-7) [2].

### 6.3. Data Collection and Response Rates

Of the 249 scholars, 27 emails bounced back or were coded as spam by mail servers. Survey link successfully reached *N* = 222 participants of which *n* = 115 (52%) completed the survey. To assess for missing data, Qualtrics reports were generated indicating 100% completion of survey.

### 6.4. Data Analysis

The respondents' age groups, years of work experience, career stage, and mentor scores were variables with highly skewed distributions (Table 1). Therefore, nonparametric statistics were used to analyze relationships and group differences. A scatter plot detected no concentration of missing data across multiple variables. The range of survey scores was assessed using scale scores from low to high for mentoring (1 to 6) and political skill (1 to 7). Mann-Whitney tests were conducted to assess differences between Ph.D. and DNP groups across multiple variables. The potential effect modifiers in this study were identified as mentoring contract, graduation status, participation in leadership development, age, work experience, and career stage (i.e., novice, competent, and proficient) [39]. To determine whether these variables were acting as modifiers, the Kruskal-Wallis statistic was used to assess differences in the ranks of scores by career stage, age group, and years of experience. The odds ratio was used to determine any likelihood that differences between graduation status and participation in a leadership or fellowship program impacted political skills scores. To determine the strengths of correlations of protégés' perceived shared traits with mentors, Spearman's Rho was calculated.

## 7. Results

This study examined the mentorship experiences of doctoral nurses and students to (a) describe the mentoring functions provided and experienced by the protégé; (b) characterize the associations between the mentoring elements and development of political skills for the protégé; and (c) compare and contrast mentored to nonmentored doctoral nurses with mentoring functions that influence political skill development.

Of the 115 respondents, 74 (64%) reported having a mentor and 41 (35.6%) reported not having a mentor (Table 2). The protégés responded that eighty-one percent of the mentors worked in an academic setting with 90.6% reporting 16 or more years of work experience. The most common terminal degree for the mentors described by protégés was a doctoral degree (82.4%). An informal mentoring relationship was described by 62% and formal mentoring by 35% of the protégés, but only 25% (*n* = 19) established a mentoring contract. The length of time of the mentoring relationship ranged from “<2 years (40.5%)” to “6+ years (10.8%)” with the most often being “3–5 years” (48.6%). Protégés reported the most common mentoring phases were “cultivating (51.4%)” (i.e., knowing each other well, close relationship, and both psychosocial and career support provided) and “redefinition (23.0%)” (i.e., moving into a peer relationship, mutually supporting each other). The most frequent forms of communication were weekly email (56%), followed by monthly phone call (24%) or formal 1 : 1 meeting (28%), and finally social networking on a quarterly basis (41%) outside of work/school. The similarity ratings between mentor and protégé were rated using a 5-point metric: 1 =* strongly disagree* to 5 =* strongly agree*. Based on results of Spearman's Rho, the strongest correlations indicating moderate effect at the significance level of *p* < 0.01 were intellectual/innovative thinking (.41), ambition (.35), approach to work (.36), social capital (.35), problem solving (.37), and values about life in general (.30) (Digital Supplemental Content, available online at http://dx.doi.org/10.1155/2016/3975634). The interpersonal relationship and mutual selection of mentoring seem important with 62% having an informal mentor, along with the reported high similarities between protégé and mentor. Mentoring contracts to establish clear development goals for protégés were low at 25% and the cultivating phase is the most active phase of the mentoring relationship as reported by protégés.

The highest mentoring subscale score was role modeling (*M* = 5.53) while the lowest score was friendship (*M* = 3.60) (Table 3). There were no significant differences in mentoring functions by type of degree (Ph.D. or DNP) as demonstrated by Mann-Whitney test (*U* = 0.35, *p* > 0.73; data not shown). In addition, there were no significant differences by type of degree (Ph.D. or DNP) for total political skill score as demonstrated by Mann-Whitney test (*U* = 0.32, *p* > 0.76; data not shown).

### 7.1. Political Skill

The self-perceived levels of political skill were as follows: 64% gave themselves ratings in the “average” political skill range and another 35.7% considered themselves to have “high” political skills (Table 4).

### 7.2. Mentoring and Political Skill

The Kruskal-Wallis rank-based test was utilized to assess for differences for variables identified as potentially modifying political skill scores but was nonsignificant for career stage (*p* = 0.06), age group (*p* = 0.83), and years of work experience (*p* = 0.09). The odds ratio found no modifying effect for graduation status (*p* = 0.12) or participation in a leadership or fellowship program (*p* = 0.22) to political skill scores. The Mann-Whitney test to assess between mentored and nonmentored nurses for political skill scores was significant (*U* = 3.35, *p* < 0.001). The Spearman Rho subscale correlations between mentoring functions and political skill are displayed in Table 5. The total mentoring score showed significance at *p* < 0.05 to total political skill and moderate effect on the networking ability subscale at *p* < 0.01. The mentoring functions of advocacy, career development facilitation, learning facilitation, and friendship were found to correlate significantly with total political skill scores at *p* < 0.05. The mentoring function of learning facilitation correlated with the political skill subscales of social astuteness and networking ability. The mentoring functions personal and emotional guidance, coaching, advocacy, career development, strategies and systems advice, and friendship correlated with the networking ability subscale. There were no significant correlations with the apparent sincerity or interpersonal influence subscale.

## 8. Discussion

This research study describes the mentoring elements provided to Ph.D. and DNP nurses in the protégé-mentor relationship and their correlations to political skill development. To recap, the response rate was 52% (*n* = 115) of which 86 were Ph.D. and 29 were DNPs, 87% female (*n* = 100), with 64% (*n* = 74) mentored. For both participants who either have earned or are candidates for a doctoral degree, protégés reported having more than 10 years of work experience and self-identified at the proficient or expert career level. The cadre of Ph.D. and DNP Jonas Nurse Scholars were highly experienced with 59% (*n* = 68) participating in a leadership or fellowship program, yet benefits from mentoring were found to support political skill development and impact of prior (nonmentored) programs was negligible. There is limited research to assess the mentoring experience of doctoral nurses for psychosocial and career support utilizing a validated mentoring survey. The career mentoring functions most often provided were coaching and advocacy closely followed by career development facilitation and strategies and systems advice. While* friendship *was the least often reported mentoring function, it was found to correlate to political skill. Friendship may have been fostered by the high rate of similarities between mentors and protégés, allowing for an open and safe learning environment leading to a positive interpersonal exchange. Advice without friendship feels cold, knowledge without support is sterile, and candor without care is harsh [40] indicating the importance of psychosocial support akin to friendship in the mentoring relationship.

Consistent with the literature, mentored individuals benefit from the mentoring relationship and in this study this was evidenced by higher levels of political skill. While nurses were all highly capable and experienced, mentoring enhanced the protégé's networking ability to gain access to social networks and increase their visibility [6, 7]. Political skill research conducted in organization psychology has found that mentoring is a mechanism that supports learning facilitation [41, 42]. An interesting finding is that while role modeling had the highest reported mean score, it did not correlate to political skill. In addition, the interpersonal influence subscale of political skill did not correlate to any of the mentoring functions. A potential rationale for this finding is the limited opportunity protégés had to observe the mentor in action since email, conference calls, and formal 1 : 1 meetings were the forms of communication most often reported. Utilizing a mentoring contract could assure that professional development goals are set, social networks expanded, and opportunities formally requested to observe mentors in action (i.e., leading in committees or interprofessional teams). The majority of mentors represented academia at 76% (*n* = 56) so consideration could be given to diversifying settings for mentoring relationships. The contextual learning that occurs in hospital, federal agency, private/nonprofit organization, or community health setting differs as a result of organizational culture. The nuances of interpersonal influence, social astuteness, and networking ability might be tied to mentoring functions if these were more specifically contracted for and experienced in diverse settings.

## Supplementary Material

Perceived similiarity between mentors-proteges is a better predictor of protégé's satisfaction with and support received from their mentors than is demographic similiarity. In fact deep-level similarity in attitudes, beliefs, and values as well as shared experiences like educational background are likely to influence the mentoring relationship.

## Figures and Tables

**Table 1:** Demographic data (*N* = 115).

Variable	Category	*n*	%
Type of program	Ph.D.	86	74.8
	DNP	29	25.2

Graduated	No	65	56.5
	Yes	50	43.5

Sex	Female	100	87.0
	Male	15	13.0

Age group	25 to 34 years	24	20.9
	35 to 44 years	37	32.2
	45 to 54 years	29	25.2
	55 to 64 years	25	21.7

Race/ethnicity	Black	17	14.8
	White	81	70.4
	Hispanic, Asian, more than one race	17	14.8

Military service	No	101	87.8
	Yes	14	12.2

Current employment status	Working full time	63	54.8
	Working part time and a full time student	19	16.5
	Working full time and a full time student	18	15.7
	Other	15	13.0

Work setting	Hospital	30	26.1
	Academic setting	48	41.7
	Other	30	26.1
	Not applicable	7	6.1

Years of work experience	Under 4 years	11	9.6
	4–6 years	13	11.3
	7–10 years	15	13.0
	11–15 years	19	16.5
	16+ years	57	49.6

Self-described level of expertise	Novice	3	2.6
	Advanced beginner	8	7.0
	Competent	18	15.7
	Proficient	26	22.6
	Expert	60	52.2

Participated in a leadership program	No	47	40.9
	Yes	68	59.1

**Table 2:** Mentoring demographics (*N* = 115).

Variable	Category	*n*	%
Mentoring status	Yes, I have a mentor	74	64.4
	No mentor	41	35.6

Description of relationship	Informal mentoring relationship	46	62.2
	Formal mentoring relationship	26	35.1
	Mentoring program	2	2.7

Mentoring contract	Yes	19	25.7
	No	55	74.3

Length of mentoring	1-2 years	30	40.5
	3–5 years	36	48.6
	6+ years	8	10.8

Mentoring phase	Initiation phase	10	13.5
	Cultivating phase	38	51.4
	Separation phase	9	12.2
	Redefinition phase	17	23.0

Mentors work environment	Hospital	6	8.1
	Academic setting	60	81.1
	Other	8	10.8

Mentor's years of experience	1–5 years	2	2.7
	6–15 years	5	6.8
	16+	67	90.6

Mentor's highest degree	Masters (MBA, M.P.H., M.S.)	4	5.4
	Doctoral (Ph.D., EdD, J.D.)	61	82.4
	DNP	4	5.4
	M.D.	5	6.8

Mentor's professional position	Supervisor/Manager	2	2.7
	Director	6	8.1
	Vice President/Sr. Vice President	2	2.7
	Assistant or Associate Professor	19	27.7
	Tenured Professor	29	39.2
	Dean	8	10.8
	Other	8	10.8

**Table 3:** Mentoring functions by rank order of mean scores for psychosocial and career subscale (scale range 1 to 6).

Scale scores	*n*	Number of items	Mean	SD	Low score	High score
*Psychosocial mentoring functions*						
Role modeling	74	4	**5.53**	0.81	2.00	6.00
Learning facilitation	74	6	**5.30**	0.79	3.00	6.00
Personal and emotional guidance	74	8	**4.85**	1.20	1.13	6.00
Friendship	74	2	**3.60**	1.36	1.00	6.00
*Career mentoring functions*						
Coaching	74	4	**5.17**	1.01	2.00	6.00
Advocacy	74	4	**5.11**	1.07	1.50	6.00
Career development facilitation	74	4	**5.06**	0.94	1.75	6.00
Strategies and systems advice	74	4	**4.96**	1.18	1.50	6.00

**Table 4:** Self-perceived level of political skill (*N* = 115).

Skill level (ratings)	*n*	%
Low skill (1 or 2)	0	0.0
Average skill (3, 4, or 5)	74	64.3
High skill (6 or 7)	41	35.7

*Note*. Ratings were based on a 7-point scale: 1 = *strongly disagree* to 7 = *strongly agree*. The scale of low, average, and high skill is based on Ferris [32].

**Table 5:** Correlations between political skill and mentoring scores using Spearman's Rho (*N* = 74).

Mentored	Political skill total score	Social astuteness subscale	Interpersonal influence subscale	Networking ability subscale	Apparent sincerity subscale
Mentoring total score	.264^*∗*^	.150	.134	.351^*∗∗*^	.82
Mentoring subscale:					
(i) Personal and emotional guidance	.158	.056	.059	.265^*∗*^	.091
(ii) Coaching	.209	.124	.135	.292^*∗*^	.099
(iii) Advocacy/sponsorship	.242^*∗*^	.178	.121	.280^*∗*^	−.061
(iv) Career development facilitation	.336^*∗*^	.179	.158	.396^*∗∗*^	.126
(v) Role modeling	−.018	−.116	−.037	.101	.033
(vi) Strategies and systems advice	.221	.124	.117	.265^*∗*^	.101
(vii) Learning facilitation	.293^*∗*^	.241^*∗*^	.205	.292^*∗*^	.149
(viii) Friendship	.283^*∗*^	.084	.158	.408^*∗∗*^	.163

^*∗*^Significant at *p* < 0.05, small effect size.

^*∗∗*^Significant at *p* < 0.01, moderate effect size.

## Additional Points

*Limitations*. This is the 1st study to utilize established validated tools to assess mentoring and political skill among nurses with either candidacy for or an earned doctoral degree. The survey instruments utilized provided objective assessments of the internal psychological states and perceptions of political skill but were not used in this study for measuring behavioral effectiveness [42], which could be captured by observer assessments of participants. One of the limitations of this research is the self-reporting bias that occurs as an individual's self-perception may be influenced by underlying motives of not wanting to appear lacking in a particular skill. Generalizability is limited because of the homogeneity of the protégés who were all Jonas Scholars and were similar in race, age, years of experience, and career stage. Nonresponse bias might exist if participant perspectives differed from those who did not participate but such comparative data were not available. The mentor's level of political or leadership skill was not assessed and is a limitation in this study.

*Recommendations*. The complex healthcare environment places high demand for well-prepared nurse leaders possessing high levels of political skills, enabling them to know precisely how to act with sincerity in social situations and use power unobtrusively [2, 43]. Political skills are essential for nurses because low political skill places them at a disadvantage for gaining power, information, and resources via social networks [4, 38, 43, 44]. As protégés, nurses at the doctoral level benefit from the interpersonal mentoring relationship to help cultivate maturity and establish set goals for career advancement, sponsorship, and the development of political skill to advance organizational and personal goals. As a protégé at this high level of preparation, it is important to develop mentoring intelligence and embrace the concept of mentoring as a professional and personal development opportunity.

*Implications for Future Research*. Future researchers should continue to explore (a) the status of political skill among nurse leaders; (b) political skill as a catalyst of leadership influence for nurses serving leadership roles and the outcomes of that influence; (c) assessment of protégé-mentor dyads to compare and contrast mentoring functions under varied pairings; (d) inclusion of supervisor, observer, and follower feedback for objective evaluation of political skill; and (e) exploration of the mentoring functions provided to Ph.D. and DNP nurse candidates using longitudinal design to measure the effectiveness over time.

*Summary*. Using standardized political skill and mentoring assessment instruments, the results of this study establish for the 1st time that both Ph.D. and DNP doctoral nurses benefit from mentoring to support political skill development. Organizational leaders would benefit from having these highly skilled and educated nurses serve roles in leadership, research, and practice transformation. Nursing organizations and researchers need to take responsibility in supporting political skill development of the next cadre of leaders to enhance the personal and professional growth of leadership competencies. High level political skills are essential to enhance one's networking ability and interpersonal influence and to develop social capital to achieve both organizational and personal career goals.
