# Supplementary material for: Mentoring Nurses in Political Skill to Navigate Organizational Politics
Source: Nurs Res Pract. 2016 Sep 29;2016:3975634. doi: 10.1155/2016/3975634 (PMC5061956; doi:10.1155/2016/3975634)
Supplement: Supplementary file 1 — Perceived similiarity between mentors-proteges is a better predictor of protégé's satisfaction with and support received from their mentors than is demographic similiarity. In fact deep-level similarity in attitudes, beliefs, and values as well as shared experiences like educational background are likely to influence the mentoring relationship. [file 3975634.f1.pdf]

Manuscript: 3975634   Mentoring Nurses in Political Skill to Navigate Organizational Politics

Digital Supplemental: Shared Similarity in the Mentor-Protégé Relationship

Spearman Correlations for the Nine Mentor/ Protégé Similarity Ratings with the Total Mentoring based on a 5-point metric: 1 = Strongly Disagree to 5 = Strongly Agree.

Score (n = 74)

Mentoring Similarity	Mentoring Score
Intellectual /Innovative thinking	.38 ****
Personality	.46 ****
Ambition	.38 ****
Approach to work or assignments	.58 ****
Communication Style	.59 ****
Social Capital	.35 ***
Problem solving skills	.59 ****
Values about life in general	.37 ****
Values of work-personal life balance	.40 ****

. *** $p < .005$. **** $p < .001$.
